# Supplementary material for: Effect of Alkaline Conditions on Forming an Effective G4.0 PAMAM Complex with Doxorubicin
Source: Pharmaceutics. 2023 Mar 8;15(3):875. doi: 10.3390/pharmaceutics15030875 (PMC10057121; doi:10.3390/pharmaceutics15030875)
Supplement: Supplementary file 1 [file pharmaceutics-15-00875-s001.zip › pharmaceutics-2209178-supplementary.pdf]

Supplementary material

Effect of alkaline conditions on forming an effective G4.0 PAMAM complex with doxorubicin.

Magdalena Szota[1], Barbara Jachimska[1*]

[1]Jerzy Haber Institute of Catalysis and Surface Chemistry Polish Academy of Sciences, Krakow, Poland

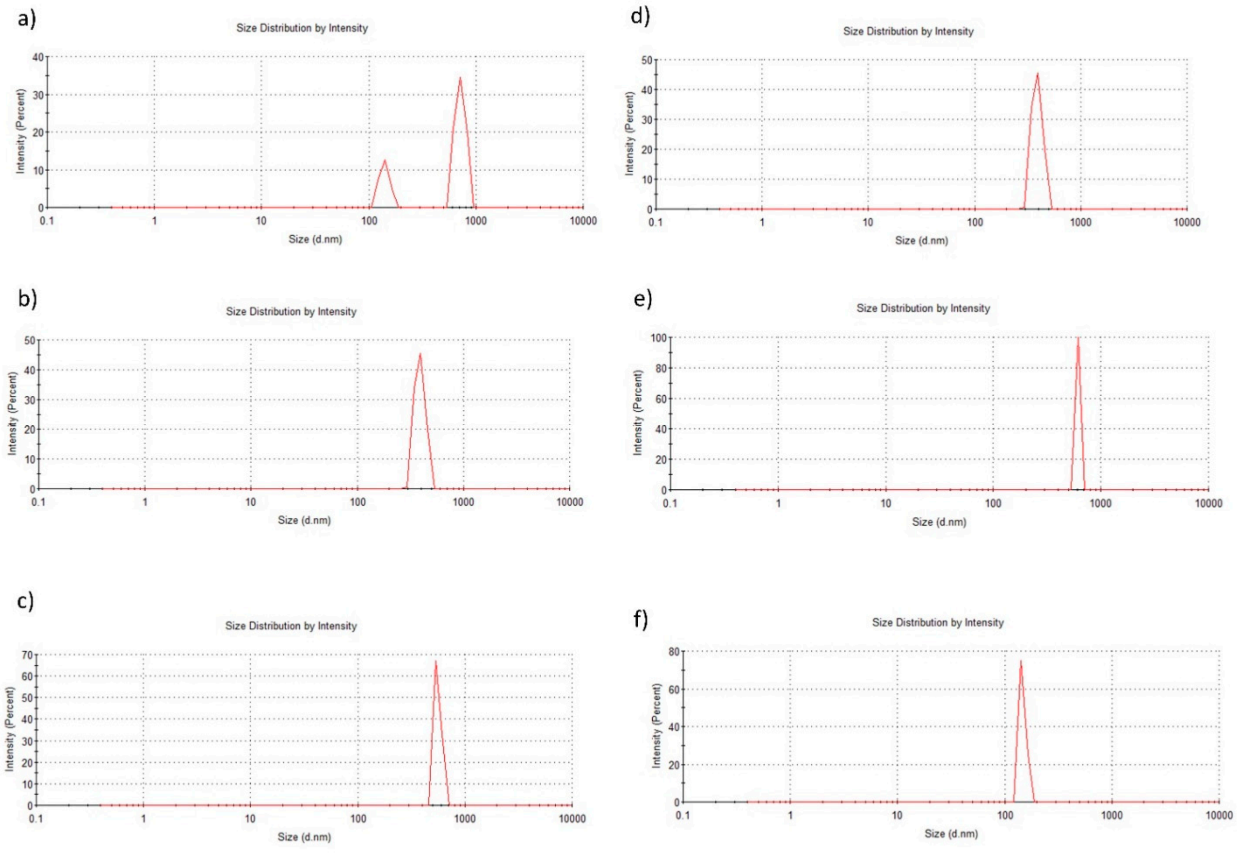

Figure S1. Particle size distribution of G4.0 PAMAM-DOX complexes by intensity as a function of pH of formation for molar ratio 1:6: a) 9.0; b) 9.5; c) 10.0 and the initial molar ratio of components: d) 1:6; e) 1:12; f) 1:24 at pH 9.5.
